# Supplementary material for: Machine Learning Models Combined with Virtual Screening and Molecular Docking to Predict Human Topoisomerase I Inhibitors
Source: Molecules. 2019 Jun 4;24(11):2107. doi: 10.3390/molecules24112107 (PMC6601036; doi:10.3390/molecules24112107)
Supplement: Supplementary file 1 [file molecules-24-02107-s001.pdf]

Supporting Information

for

Machine Learning Models Combined with Virtual Screening and Molecular Docking to Predict Human Topoisomerase I Inhibitors

Bingke Li[1,2], Xiaokang Kang[1], Dan Zhao[1], Yurong Zou[1], Xudong Huang[1], Jiexue Wang[1,*], Chenghua Zhang[3,*]

[1] Institute of Functional Molecules, College of Chemistry and Life Science, Chengdu Normal University, Chengdu 611130, China; KXK2799956460@163.com (X.K.); audreyzhaodan@163.com (D.Z.); zyrlia1018@163.com (Y.Z.); hxd1997116@163.com (X.H.)

[2] School of Biological and Chemical Engineering, Nanyang Institute of Technology, Nanyang 473004; libingke86@126.com (B.L.)

[3] School of Basic Medical Sciences, North Sichuan Medical College, Nanchong 637000

* Corresponding authors. E–mail addresses: xueer0702@163.com (J.W.); zchua@nsmc.edu.cn (C.Z.)

Table of Contents

	Caption	Page
Table S1	The effect of the different values of Ntree (100 ≤ Ntree ≤ 3000) on the final OOB PERs of RF within the training set and the testing set for the subject of Top1.	1
Table S2	Relatively important 63 descriptors identified by the optimal RF model for the prediction of Top1is and non-Top1is.	5
Table S3	Relatively important 63 descriptors identified by SVM for the prediction of Top1is and non-Top1is.	7
Table S4	Relatively important 37 descriptors identified by C4.5 DT for the prediction of Top1is and non-Top1is.	9
Table S5	67 molecules with RF scores greater than 0.7.	11
Table S6	All molecular descriptors used in this work.	13
Figure S1	The three actual non-Top1is mispredicted as Top1is.	20
Figure S2	The three actual Top1is mispredicted as non-Top1is (three out of eleven).	20
Figure S3	The visualized distributions of compounds. (A) in the external validation set (55 compounds), (B) in the date set of molecules singled out by virtual screening with RF scores greater than 0.7 (67 compounds).	21
Figure S4	The molecular weight distributions of compounds. (A) in the external validation set (55 compounds), (B) in the date set of molecules singled out by virtual screening with RF scores greater than 0.7 (67 compounds).	22

Table S1. The effect of the different values of N_{tree} ($100 \leq N_{tree} \leq 3000$) on the final OOB PERs of RF within the training set and the testing set for the subject of Top1.

Value of N_{tree} (the initial step-size is equal to **100**)	Final OOB PER % (within the training set)	Final OOB PER % (within the testing set)
100	9.26879501	3.29218102
**200**	**8.44490242**	**2.88065839**
300	9.06282234	3.08641982
400	8.65087509	3.08641982
**500**	**8.95983505**	**2.88065839**
**600**	**8.95983505**	**2.88065839**
700	8.95983505	3.08641982
800	9.06282234	3.08641982
900	8.75386238	3.29218102
1000	8.65087509	3.29218102
1100	8.54788876	3.08641982
1200	8.65087509	3.08641982
1300	8.44490242	3.08641982
1400	8.34191608	3.08641982
1500	8.23892879	3.08641982
**1600**	**8.44490242**	**2.88065839**
1700	8.54788876	3.08641982
1800	8.65087509	3.08641982
1900	8.75386238	3.08641982
2000	8.54788876	3.08641982
2100	8.54788876	3.08641982
2200	8.75386238	3.08641982
2300	8.54788876	3.08641982
2400	8.54788876	3.08641982
2500	8.54788876	3.08641982
2600	8.44490242	3.08641982
2700	8.54788876	3.08641982
2800	8.44490242	3.08641982
2900	8.44490242	3.08641982
3000	8.54788876	3.08641982
Value of N_{tree} (the initial step-size is equal to **10**)	Final OOB PER % (within the training set)	Final OOB PER % (within the testing set)
100	9.26879501	3.29218102
110	8.75386238	3.29218102
120	8.34191608	3.29218102
130	8.23892879	3.08641982

140	8.75386238	3.29218102
150	8.54788876	3.29218102
160	8.85684872	3.29218102
170	8.54788876	3.08641982
**180**	**8.34191608**	**2.88065839**
**190**	**8.44490242**	**2.88065839**
**200**	**8.44490242**	**2.88065839**
**210**	**8.44490242**	**2.88065839**
**220**	**8.75386238**	**2.88065839**
**230**	**9.16580868**	**2.88065839**
240	8.85684872	3.08641982
250	8.95983505	3.08641982
260	8.95983505	3.08641982
270	8.95983505	3.08641982
280	9.06282234	3.08641982
290	8.95983505	3.08641982
300	9.06282234	3.08641982
310	8.75386238	3.49794245
320	8.95983505	3.29218102
330	9.06282234	3.49794245
340	8.95983505	3.49794245
350	8.75386238	3.49794245
360	9.16580868	3.29218102
370	8.85684872	3.29218102
380	8.95983505	3.49794245
390	8.54788876	3.29218102
400	8.65087509	3.08641982
410	8.44490242	3.29218102
420	8.54788876	3.29218102
430	8.65087509	3.08641982
440	8.75386238	3.08641982
450	8.75386238	3.08641982
460	8.85684872	3.29218102
470	8.75386238	3.29218102
**480**	**8.95983505**	**2.88065839**
**490**	**8.85684872**	**2.88065839**
**500**	**8.95983505**	**2.88065839**
**510**	**9.06282234**	**2.88065839**
**520**	**9.06282234**	**2.88065839**
**530**	**9.06282234**	**2.88065839**
**540**	**9.16580868**	**2.88065839**

**550**	**9.26879501**	**2.88065839**
**560**	**9.16580868**	**2.88065839**
570	9.16580868	3.08641982
580	9.06282234	3.08641982
590	9.06282234	3.08641982
**600**	**8.95983505**	**2.88065839**
610	8.85684872	3.08641982
620	8.95983505	3.08641982
630	8.85684872	3.08641982
640	8.95983505	3.08641982
650	8.85684872	3.08641982
660	8.75386238	3.08641982
670	9.06282234	3.08641982
680	8.95983505	3.08641982
690	8.85684872	3.08641982
700	8.95983505	3.08641982
1500	8.23892879	3.08641982
1510	8.23892879	3.08641982
1520	8.23892879	3.08641982
1530	8.23892879	3.08641982
1540	8.34191608	3.08641982
1550	8.34191608	3.08641982
1560	8.34191608	3.08641982
1570	8.44490242	3.08641982
**1580**	**8.34191608**	**2.88065839**
**1590**	**8.44490242**	**2.88065839**
**1600**	**8.44490242**	**2.88065839**
**1610**	**8.54788876**	**2.88065839**
1620	8.65087509	3.08641982
1630	8.54788876	3.08641982
1640	8.54788876	3.08641982
1650	8.65087509	3.08641982
1660	8.44490242	3.08641982
1670	8.54788876	3.08641982
1680	8.65087509	3.08641982
1690	8.65087509	3.08641982
1700	8.54788876	3.08641982
Value of N_{tree} (the initial step-size is equal to **1**)	Final OOB PER % (within the training set)	Final OOB PER % (within the testing set)
170	8.54788876	3.08641982
171	8.54788876	3.08641982

172	8.44490242	3.08641982
173	8.54788876	3.08641982
174	8.54788876	3.08641982
175	8.44490242	2.88065839
176	8.54788876	2.88065839
177	8.54788876	2.88065839
178	8.44490242	2.88065839
179	8.34191608	2.88065839
180	8.34191608	2.88065839
**181**	**8.23892879**	**2.88065839**
**182**	**8.23892879**	**2.88065839**
**183**	**8.23892879**	**2.88065839**
**184**	**8.23892879**	**2.88065839**
185	8.54788876	2.88065839
186	8.54788876	2.88065839
187	8.54788876	2.88065839
188	8.65087509	2.88065839
189	8.54788876	2.88065839
190	8.44490242	2.88065839
1570	8.44490242	3.08641982
1571	8.44490242	3.08641982
1572	8.34191608	3.08641982
1573	8.44490242	3.08641982
1574	8.44490242	3.08641982
1575	8.34191608	3.08641982
1576	8.34191608	2.88065839
1577	8.34191608	2.88065839
1578	8.34191608	2.88065839
1579	8.34191608	2.88065839
1580	8.34191608	2.88065839
1581	8.34191608	2.88065839
1582	8.34191608	2.88065839
1583	8.44490242	2.88065839
1584	8.44490242	2.88065839
1585	8.44490242	2.88065839
1586	8.44490242	2.88065839
1587	8.44490242	2.88065839
1588	8.44490242	2.88065839
1589	8.44490242	2.88065839
1590	8.44490242	2.88065839

Table S2. Relatively important 63 descriptors identified by the optimal RF model for the prediction of Top1is and non-Top1is.

Descriptor	Ranking	Description
Rugty	1	Molecular rugosity
Tcent	2	Centric Index
S(27)	3	Atom-type Estate sum for : C ::
S(38)	4	Atom-type Estate sum for :N:-
${}^{6}\chi^{v}_{CH}$	5	Valence molecular connectivity Chi indices for cycles of 6 atoms
Nring	6	Numbers of rings
Hiwpl	7	Hydrophilic Intery Moment
S(22)	8	Atom-type Estate sum for >CH-
${}^{4}\chi_{PC}$	9	Simple molecular connectivity Chi indices for path/cluster
${}^{6}\chi_{CH}$	10	Simple molecular connectivity Chi indices for cycles of 6 atom
S(35)	11	Atom-type Estate sum for :N:
${}^{3}\kappa_{\alpha}$	12	Kappa alpha indices for three boned fragments
$Q_{O, Max}$	13	Most positive charge on O atoms
Nrot	14	Number of rotatable bonds
$Q_{N, Min}$	15	Most negative charge on N atoms
Tiwie	16	Information Weiner
S(26)	17	Atom-type Estate sum for : C:-
$Q_{N, SS}$	18	Sum of squares of charges on N atoms
${}^{3}\chi_{P}$	19	Simple molecular connectivity Chi indices for path order 3
${}^{3}\kappa$	20	Molecular shape Kappa indices for three boned fragments
S(21)	21	Atom-type Estate sum for : CH : (aromatic)
${}^{5}\chi^{v}_{CH}$	22	Valence molecular connectivity Chi indices for cycles of 5 atoms
Hiwpb	23	Hydrophobic Intery Moment
$A_{Q, max}$	24	Most positive charge in a molecule
S(41)	25	Atom-type Estate sum for -O-
$Q_{C, Max}$	26	Most positive charge on C atoms
S(10)	27	Atom-type H Estate sum for :C**H**: (sp^2, aromatic)
S(9)	28	Atom-type H Estate sum for =C**H**- (sp^2)
phi	29	Kier molecular flexibility index
dis1	30	Length vectors (longest distance)
S(25)	31	Atom-type Estate sum for =C<
S(34)	32	Atom-type Estate sum for =N-
$Q_{C, Min}$	33	Most negative charge on C atoms
Rpc	34	Relative positive charge
$Q_{O, SS}$	35	Sum of squares of charges on O atoms
Sapcw	36	Sum of charge weighted solvent accessible surface areas of positively charged atoms

$Q_{O, Min}$	37	Most negative charge on O atoms
Tbala	38	Balaban Index
S(40)	39	Atom-type Estate sum for =O
Mac	40	Mean absolute charge
$A_{Q, min}$	41	Most negative charge in a molecule
Shpb	42	Hydrophobic region
$Q_{N, Max}$	43	Most positive charge on N atoms
S(13)	44	Atom-type H Estate sum for C**H** $_n$ (unsaturated)
Nnitro	45	Count of N atoms
$Q_{H, Min}$	46	Most negative charge on H atoms
Svpc	47	Sum of van der Waals surface areas of positively charged atoms
Tradi	48	PetitJohn R2 Index
$^5\chi_{CH}$	49	Simple molecular connectivity Chi indices for cycles of 5 atom
$Q_{C, SS}$	50	Sum of squares of charges on C atoms
dis2	51	Length vectors (longest third atom)
Gloty	52	Molecular globularity
PSA	53	Polar molecular surface area
Svpcw	54	Sum of charge weighted van der Waals surface areas of positively charged atoms
$^2\kappa$	55	Molecular shape Kappa indices for two boned fragments
$^4\chi^v_{PC}$	56	Valence molecular connectivity Chi indices for path/cluster
Svnc	57	Sum of van der Waals surface areas of negatively charged atoms
$^3\chi^v_P$	58	Valence molecular connectivity Chi indices for path order 3
dis3	59	Length vectors (4th atom)
S(1)	60	Atom-type H Estate sum for -O**H**
Capty	61	Capacity factor
$Q_{H, SS}$	62	Sum of squares of charges on H atoms
Hiwpa	63	Amphiphilic Moment

Table S3. Relatively important 63 descriptors identified by SVM for the prediction of Top1is and non-Top1is.

Descriptor	Ranking	Description
$^{5}\chi_{CH}$	1	Simple molecular connectivity Chi indices for cycles of 5 atom
Rugty	2	Molecular rugosity
Tcent	3	Centric Index
S(10)	4	Atom-type H Estate sum for :C**H**: (sp^{2}, aromatic)
$Q_{N, Min}$	5	Most negative charge on N atoms
S(25)	6	Atom-type Estate sum for =C<
$Q_{C, Max}$	7	Most positive charge on C atoms
$^{6}\chi^{v}_{CH}$	8	Valence molecular connectivity Chi indices for cycles of 6 atoms
Tiwie	9	Information Weiner
S(27)	10	Atom-type Estate sum for :C::
S(26)	11	Atom-type Estate sum for :C:-
Rpc	12	Relative positive charge
S(37)	13	Atom-type Estate sum for -N<< (NO_2)
$Q_{N, Max}$	14	Most positive charge on N atoms
Hiwpa	15	Amphiphilic Moment
Hiwpb	16	Hydrophobic Intery Moment
S(20)	17	Atom-type Estate sum for =CH-
$Q_{H, Min}$	18	Most negative charge on H atoms
S(21)	19	Atom-type Estate sum for :CH: (aromatic)
S(34)	20	Atom-type Estate sum for =N-
S(41)	21	Atom-type Estate sum for -O-
S(36)	22	Atom-type Estate sum for >N-
$^{6}\chi_{CH}$	23	Simple molecular connectivity Chi indices for cycles of 6 atom
Capty	24	Capacity factor
S(16)	25	Atom-type Estate sum for -CH_3
μ	26	Molecular dipole moment
$Q_{H, Max}$	27	Most positive charge on H atoms
S(9)	28	Atom-type H Estate sum for =C**H**- (sp^{2})
Mpc	29	Mean of positive charges
Hiwpl	30	Hydrophilic Intery Moment
nring	31	Numbers of rings
S(18)	32	Atom-type Estate sum for >CH_2
Rnc	33	Relative negative charge
Mnc	34	Mean of negative charges
S(13)	35	Atom-type H Estate sum for $C\mathbf{H}_n$ (unsaturated)
$Q_{C, Min}$	36	Most negative charge on C atoms

S(38)	37	Atom-type Estate sum for :N:-
$^{5}\chi^{v}_{CH}$	38	Valence molecular connectivity Chi indices for cycles of 5 atoms
$^{4}\chi^{v}_{PC}$	39	Valence molecular connectivity Chi indices for path/cluster
Mac	40	Mean absolute charge
Shpb	41	Hydrophobic region
Tradi	42	PetitJohn R2 Index
dis1	43	Length vectors (longest distance)
Gloty	44	Molecular globularity
S(32)	45	Atom-type Estate sum for :NH:
S(56)	46	Atom-type Estate sum for -S-
$^{4}\chi_{PC}$	47	Simple molecular connectivity Chi indices for path/cluster
S(2)	48	Atom-type H Estate sum for =N**H**
S(60)	49	Atom-type Estate sum for -Cl
S(40)	50	Atom-type Estate sum for =O
$A_{Q, min}$	51	Most negative charge in a molecule
S_{car}	52	Sum of Estate indices of carbon atoms
nnitro	53	Count of N atoms
S(14)	54	Atom-type H Estate sum for $C\mathbf{H}_n$ (aromatic)
$A_{Q, max}$	55	Most positive charge in a molecule
S(35)	56	Atom-type Estate sum for :N:
Sanc	57	Sum of solvent accessible surface areas of negatively charged atoms
S(42)	58	Atom-type Estate sum for :O:
S(5)	59	Atom-type H Estate sum for >N**H**
$Q_{O, Max}$	60	Most positive charge on O atoms
nsulph	61	Count of S atoms
S(29)	62	Atom-type Estate sum for $-NH_2$
S(4)	63	Atom-type H Estate sum for $-N\mathbf{H}_2$

Table S4. Relatively important 37 descriptors identified by C4.5 DT for the prediction of Top1is and non-Top1is.

Descriptor	Ranking	Description
S(27)	1	Atom-type Estate sum for :C::
nring	2	Numbers of rings
S(32)	2	Atom-type Estate sum for :NH:
Gloty	4	Molecular globularity
nrot	4	Number of rotatable bonds
S(42)	4	Atom-type Estate sum for :O:
$Q_{H, Min}$	7	Most negative charge on H atoms
S(5)	7	Atom-type H Estate sum for >N**H**
S(2)	7	Atom-type H Estate sum for =N**H**
$Q_{O, Max}$	7	Most positive charge on O atoms
dis3	11	Length vectors (4th atom)
S(31)	11	Atom-type Estate sum for >NH
nhev	11	Count of heavy atoms
$^{0}\chi^{v}$	11	Valence molecular connectivity Chi indices for path order 0
S(20)	11	Atom-type Estate sum for =CH-
S(33)	11	Atom-type Estate sum for ≡N
nhyd	11	Count of hydrogen atoms
$^{6}\chi^{v}_{CH}$	11	Valence molecular connectivity Chi indices for cycles of 6 atoms
Hiwpl	11	Hydrophilic Intery Moment
Svpc	20	Sum of van der Waals surface areas of positively charged atoms
$Q_{N, Min}$	20	Most negative charge on N atoms
nhet	20	Count of hetero atoms
nnitro	23	Count of N atoms
S(26)	23	Atom-type Estate sum for :C:-
Svpcw	25	Sum of charge weighted van der Waals surface areas of positively charged atoms
$Q_{H, Max}$	25	Most positive charge on H atoms
$Q_{C, Max}$	25	Most positive charge on C atoms
Hlb	28	Hydrophilic-Hydrophobic balance
Hiwpa	28	Amphiphilic Moment
S(37)	28	Atom-type Estate sum for -N<< (NO_2)
$^{5}\chi^{v}_{CH}$	31	Valence molecular connectivity Chi indices for cycles of 5 atoms
S(14)	31	Atom-type H Estate sum for C**H**$_n$ (aromatic)
S(35)	31	Atom-type Estate sum for :N:
S(22)	31	Atom-type Estate sum for >CH-
Mnc	35	Mean of negative charges
S(1)	35	Atom-type H Estate sum for -O**H**

noxy	35	Count of O atoms

Table S5. 67 molecules with RF scores greater than 0.7.

Sequence number	Name	RF score	Binding energy (kcal/mol)
1	MBX161745	0.912	-10.1
2	MBX135104	0.901	-8.9
3	MBX135061	0.878	-9.4
4	MBX135132	0.878	-8.1
5	MBX183355	0.851	-8.3
6	MBX135103	0.845	-8.5
7	MBX132963	0.829	-9
8	MBX135059	0.818	-8.7
9	MBX135110	0.818	-8.6
10	MBX135138	0.818	-8.7
11	MBX143215	0.818	-8.6
12	MBX212936	0.818	-9
13	MBX135102	0.807	-8.4
14	MBX190743	0.807	-8.6
15	MBX212932	0.807	-9
16	MBX133000	0.801	-8.9
17	MBX135121	0.796	-9.1
18	MBX161743	0.796	-8.8
19	MBX202304	0.796	-9.4
20	MBX212316	0.79	-9.8
21	MBX534671	0.79	-8.5
22	MBX135063	0.773	-8.7
23	MBX133352	0.762	-9.4
24	MBX135131	0.762	-8.1
25	MBX183350	0.762	-8.1
26	MBX534737	0.762	-9.5
27	MBX190730	0.757	-9.9
28	MBX190744	0.757	-9.6
29	MBX212938	0.757	-8.9
30	MBX135100	0.751	-8.5
31	MBX143217	0.751	-8.8
32	MBX212320	0.751	-8.2
33	MBX212862	0.751	-8.6
34	MBX212933	0.751	-8.7
35	MBX135099	0.746	-9.3
36	MBX212934	0.746	-8.8
37	MBX477559	0.746	-8.6
38	MBX135133	0.74	-7.9
39	MBX209152	0.74	-10.3

40	MBX133023	0.735	-9
41	MBX212321	0.735	-8.9
42	MBX190731	0.729	-9.9
43	MBX212244	0.729	-9.2
44	MBX212876	0.729	-8.9
45	MBX534706	0.729	-11.4
46	MBX135092	0.724	-9.6
47	MBX161741	0.724	-9.7
48	MBX212886	0.724	-8.7
49	MBX534707	0.724	-9.7
50	MBX212243	0.718	-8.1
51	MBX212883	0.718	-9.3
52	MBX212937	0.718	-8.3
53	MBX190732	0.713	-10.1
54	MBX201924	0.713	-9.1
55	MBX204808	0.713	-9.5
56	MBX209154	0.713	-8.9
57	MBX534660	0.713	-8.7
58	MBX135057	0.707	-9
59	MBX143200	0.707	-7.8
60	MBX161748	0.707	-10.2
61	MBX162127	0.707	-10.5
62	MBX183356	0.707	-8.5
63	MBX212860	0.707	-9.4
64	MBX162142	0.702	-9.6
65	MBX182993	0.702	-8.3
66	MBX212881	0.702	-9.4
67	MBX534585	0.702	-9.7

Table S6. All molecular descriptors used in this work.

		simple molecular properties(18)	class
1	W_{mol}	Molecular weight	1
2	nhyd	Count of hydrogen atoms	8
3	nhal	Count of halogen atoms	8
4	nhet	Count of hetero atoms	8
5	nhev	Count of heavy atoms	8
6	ncof	Count of F atoms	8
7	ncocl	Count of Cl atoms	8
8	ncobr	Count of Br atoms	8
9	ncoi	Count of I atoms	8
10	ncarb	Count of C atoms	8
11	nphos	Count of P atoms	8
12	nsulph	Count of S atoms	8
13	noxy	Count of O atoms	8, 5
14	nnitro	Count of N atoms	8,5
15	nring	Numbers of rings	8, 2
16	nrot	Number of rotatable bonds	8, 2
17	ndonr	Number of H-bond donors	4
18	naccr	Number of H-bond acceptors	5
		**molecular connectivity and shape(27)**	
19	$^{0}\chi$	Simple molecular connectivity Chi indices for path order 0	2
20	$^{1}\chi$	Simple molecular connectivity Chi indices for path order 1	2
21	$^{2}\chi$	Simple molecular connectivity Chi indices for path order 2	2
22	$^{3}\chi_{P}$	Simple molecular connectivity Chi indices for path order 3	2
23	$^{3}\chi_{C}$	Simple molecular connectivity Chi indices for cluster	2
24	$^{4}\chi_{PC}$	Simple molecular connectivity Chi indices for path/cluster	2
25	$^{3}\chi_{CH}$	Simple molecular connectivity Chi indices for cycles of 3 atoms	2
26	$^{4}\chi_{CH}$	Simple molecular connectivity Chi indices for cycles of 4 atom	2

27	${}^{5}\chi_{CH}$	Simple molecular connectivity Chi indices for cycles of 5 atom	2
28	${}^{6}\chi_{CH}$	Simple molecular connectivity Chi indices for cycles of 6 atom	2
29	${}^{0}\chi^{v}$	Valence molecular connectivity Chi indices for path order 0	2
30	${}^{1}\chi^{v}$	Valence molecular connectivity Chi indices for path order 1	2
31	${}^{2}\chi^{v}$	Valence molecular connectivity Chi indices for path order 2	2
32	${}^{3}\chi^{v}_{P}$	Valence molecular connectivity Chi indices for path order 3	2
33	${}^{3}\chi^{v}_{C}$	Valence molecular connectivity Chi indices for cluster	2
34	${}^{4}\chi^{v}_{PC}$	Valence molecular connectivity Chi indices for path/cluster	2
35	${}^{3}\chi^{v}_{CH}$	valence molecular connectivity Chi indices for cycles of 3 atoms	2
36	${}^{4}\chi^{v}_{CH}$	valence molecular connectivity Chi indices for cycles of 4 atoms	2
37	${}^{5}\chi^{v}_{CH}$	valence molecular connectivity Chi indices for cycles of 5 atoms	2
38	${}^{6}\chi^{v}_{CH}$	valence molecular connectivity Chi indices for cycles of 6 atoms	2
39	${}^{1}\kappa$	Molecular shape Kappa indices for one boned fragments	2
40	${}^{2}\kappa$	Molecular shape Kappa indices for two boned fragments	2
41	${}^{3}\kappa$	Molecular shape Kappa indices for three boned fragments	2
42	${}^{1}\kappa_{\alpha}$	Kappa alpha indices for one boned fragments	2
43	${}^{2}\kappa_{\alpha}$	Kappa alpha indices for two boned fragments	2
44	${}^{3}\kappa_{\alpha}$	Kappa alpha indices for three boned fragments	2
45	phi	Kier molecular flexibility index	2
		**electrotopological state(97)**	
46	S_{hev}	Sum of electrotopological state (Estate) indices of heavy atoms	3
47	S_{car}	Sum of Estate indices of carbon atoms	3
48	S_{het}	Sum of Estate indices of hetero atoms	3
49	S_{hal}	Sum of Estate indices of halogen atoms	3
50	S(1)	Atom-type H Estate sum for -O**H**	3, 4
51	S(2)	Atom-type H Estate sum for =N**H**	3,4
52	S(3)	Atom-type H Estate sum for -S**H**	3,4
53	S(4)	Atom-type H Estate sum for -N**H**$_2$	3,4

54	S(5)	Atom-type H Estate sum for > N**H**	3,4
55	S(6)	Atom-type H Estate sum for : N**H**:	3
56	S(7)	Atom-type H Estate sum for #C**H** (sp)	3
57	S(8)	Atom-type H Estate sum for =C**H**$_2$ (sp^2)	3
58	S(9)	Atom-type H Estate sum for =C**H**- (sp^2)	3
59	S(10)	Atom-type H Estate sum for :C**H**: (sp^2, aromatic)	3
60	S(11)	Atom-type H Estate sum for C**H**$_n$ X (sp^3, X= F, Cl, Br, I)	3
61	S(12)	Atom-type H Estate sum for C**H**$_n$ (Saturated)	3
62	S(13)	Atom-type H Estate sum for C**H**$_n$ (unsaturated)	3
63	S(14)	Atom-type H Estate sum for C**H**$_n$ (aromatic)	3
64	S(15)	Atom-type H Estate sum for A**H**$_n$ (not C, N, O, S)	3
65	S(16)	Atom-type Estate sum for -CH_3	3
66	S(17)	Atom-type Estate sum for =CH_2	3
67	S(18)	Atom-type Estate sum for >CH_2	3
68	S(19)	Atom-type Estate sum for ≡CH	3
69	S(20)	Atom-type Estate sum for =CH-	3
70	S(21)	Atom-type Estate sum for : CH : (aromatic)	3
71	S(22)	Atom-type Estate sum for >CH-	3
72	S(23)	Atom-type Estate sum for =C=	3
73	S(24)	Atom-type Estate sum for ≡C-	3
74	S(25)	Atom-type Estate sum for =C<	3
75	S(26)	Atom-type Estate sum for : C:-	3
76	S(27)	Atom-type Estate sum for : C ::	3
77	S(28)	Atom-type Estate sum for >C<	3
78	S(29)	Atom-type Estate sum for -NH_2	3
79	S(30)	Atom-type Estate sum for =NH	3
80	S(31)	Atom-type Estate sum for >NH	3
81	S(32)	Atom-type Estate sum for :NH:	3,5
82	S(33)	Atom-type Estate sum for ≡N	3

83	S(34)	Atom-type Estate sum for =N-	3
84	S(35)	Atom-type Estate sum for :N:	3,5
85	S(36)	Atom-type Estate sum for >N-	3
86	S(37)	Atom-type Estate sum for -N<< (NO_2)	3
87	S(38)	Atom-type Estate sum for :N:-	3,5
88	S(39)	Atom-type Estate sum for -OH	3,5
89	S(40)	Atom-type Estate sum for =O	3,5
90	S(41)	Atom-type Estate sum for -O-	3,5
91	S(42)	Atom-type Estate sum for :O:	3,5
92	S(43)	Atom-type Estate sum for -F	3
93	S(44)	Atom-type Estate sum for -SiH_3	3
94	S(45)	Atom-type Estate sum for -SiH_2-	3
95	S(46)	Atom-type Estate sum for >SiH-	3
96	S(47)	Atom-type Estate sum for >Si<	3
97	S(48)	Atom-type Estate sum for -PH_2	3
98	S(49)	Atom-type Estate sum for -PH-	3
99	S(50)	Atom-type Estate sum for >P-	3
100	S(51)	Atom-type Estate sum for ->P= (P.O)	3
101	S(52)	Atom-type Estate sum for -=P= (P. O_2)	3
102	S(53)	Atom-type Estate sum for -	3
103	S(54)	Atom-type Estate sum for -SH	3
104	S(55)	Atom-type Estate sum for =S	3,5
105	S(56)	Atom-type Estate sum for -S-	3
106	S(57)	Atom-type Estate sum for :S:	3,5
107	S(58)	Atom-type Estate sum for >S=O	3
108	S(59)	Atom-type Estate sum for >S<<	3
109	S(60)	Atom-type Estate sum for -Cl	3
110	S(61)	Atom-type Estate sum for GeH_3	3
111	S(62)	Atom-type Estate sum for -GeH_2-	3

112	S(63)	Atom-type Estate sum for >GeH-	3
113	S(64)	Atom-type Estate sum for>Ge<	3
114	S(65)	Atom-type Estate sum for -AsH_2	3
115	S(66)	Atom-type Estate sum for -AsH-	3
116	S(67)	Atom-type Estate sum for >As-	3
117	S(68)	Atom-type Estate sum for ->As=	3
118	S(69)	Atom-type Estate sum for -SeH	3
119	S(70)	Atom-type Estate sum for =Se	3
120	S(71)	Atom-type Estate sum for -Se-	3
121	S(72)	Atom-type Estate sum for :Se:	3
122	S(73)	Atom-type Estate sum for >Se=	3
123	S(74)	Atom-type Estate sum for -=Se=-	3
124	S(75)	Atom-type Estate sum for -Br	3
125	S(76)	Atom-type Estate sum for -SnH_3	3
126	S(77)	Atom-type Estate sum for -SnH_2-	3
127	S(78)	Atom-type Estate sum for >SnH-	3
128	S(79)	Atom-type Estate sum for >Sn<	3
129	S(80)	Atom-type Estate sum for -I	3
130	Twien	Weiner Index	2
131	Tcent	Centric Index	2
132	Talte	Altenburg Index	2
133	Tbala	Balaban Index	2
134	Thara	Harary Number	2
135	Tschl	Schultz Index	2
136	Tradi	PetitJohn R2 Index	2
137	Tdiam	PetitJohn D2 Index	2
138	Tbmdd	Mean Distance Index	2
139	Tpeti	PetitJohn I2 Index	2
140	Tiwie	Information Weiner	2

141	Trmsd	Balaban RMSD Index	2
142	Tigdi	Graph Distance Index	2
		**quantum chemical properties (22)**	
143	π_i	Polarizability index	6,7
144	μ	Molecular dipole moment	6
145	$Q_{H, Max}$	Most positive charge on H atoms	3,4
146	$Q_{C, Max}$	Most positive charge on C atoms	3,4
147	$Q_{N, Max}$	Most positive charge on N atoms	3,4
148	$Q_{O, Max}$	Most positive charge on O atoms	3,4
149	$Q_{H, Min}$	Most negative charge on H atoms	3, 5
150	$Q_{C, Min}$	Most negative charge on C atoms	3, 5
151	$Q_{N, Min}$	Most negative charge on N atoms	3, 5
152	$Q_{O, Min}$	Most negative charge on O atoms	3, 5
153	$A_{Q, max}$	Most positive charge in a molecule	3
154	$A_{Q, min}$	Most negative charge in a molecule	3
155	$Q_{H, SS}$	Sum of squares of charges on H atoms	3
156	$Q_{C, SS}$	Sum of squares of charges on C atoms	3
157	$Q_{N, SS}$	Sum of squares of charges on N atoms	3
158	$Q_{O, SS}$	Sum of squares of charges on O atoms	3
159	$Q_{A, SS}$	Sum of squares of charges on all atoms	3
160	Mpc	Mean of positive charges	3
161	Mnc	Mean of negative charges	3
162	Mac	Mean absolute charge	3
163	Rpc	Relative positive charge	3
164	Rnc	Relative negative charge	3
		**geometrical properties(25)**	
165	dis1	Length vectors (longest distance)	1
166	dis2	Length vectors (longest third atom)	1
167	dis3	Length vectors (4th atom)	1

168	V_{mc}	Van der Waals molecular volume	1
169	AS	Solvent accessible surface area	1
170	VS	van der Waals surface area	1
171	MS	Molecular surface area	1
172	PSA	Polar molecular surface area	6
173	Sapc	Sum of solvent accessible surface areas of positively charged atoms	4
174	Sanc	Sum of solvent accessible surface areas of negatively charged atoms	5
175	Sapcw	Sum of charge weighted solvent accessible surface areas of positively charged atoms	4
176	Sancw	Sum of charge weighted solvent accessible surface areas of negatively charged atoms	5
177	Svpc	Sum of van der Waals surface areas of positively charged atoms	4
178	Svnc	Sum of van der Waals surface areas of negatively charged atoms	5
179	Svpcw	Sum of charge weighted van der Waals surface areas of positively charged atoms	4
180	Svncw	Sum of charge weighted van der Waals surface areas of negatively charged atoms	5
181	Rugty	Molecular rugosity	6,7
182	Gloty	Molecular globularity	6,7
183	Shpl	Hydrophilic region	6
184	Shpb	Hydrophobic region	7
185	Capty	Capacity factor	6,7
186	Hlb	Hydrophilic-Hydrophobic balance	6,7
187	Hiwpl	Hydrophilic Intery Moment	6
188	Hiwpb	Hydrophobic Intery Moment	7
189	Hiwpa	Amphiphilic Moment	6,7

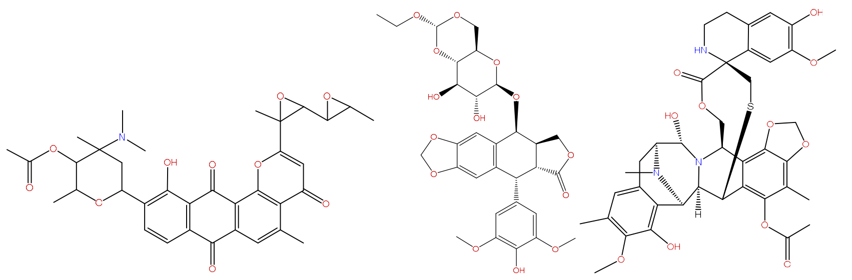

20746 33278 2936

Figure S1. The three actual non-Top1is mispredicted as Top1is.

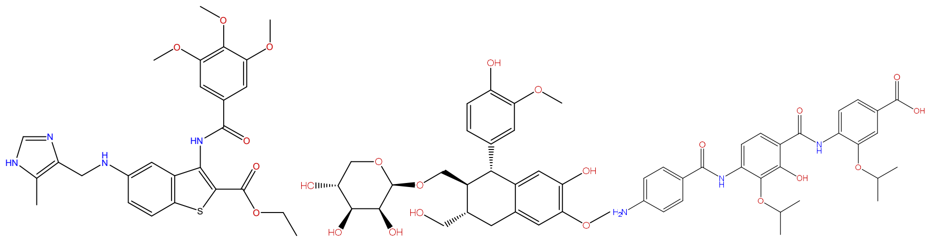

879391 837665 874128

Figure S2. The three actual Top1is mispredicted as non-Top1is (three out of eleven).

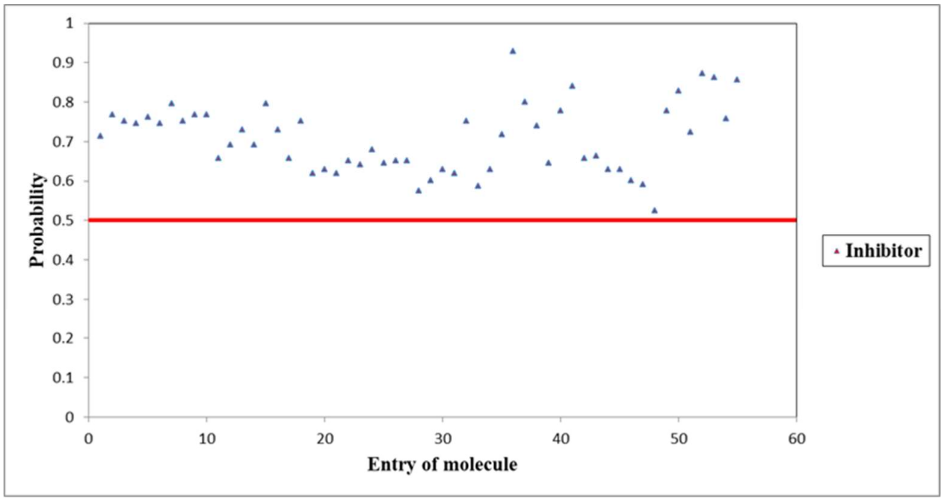

(A)

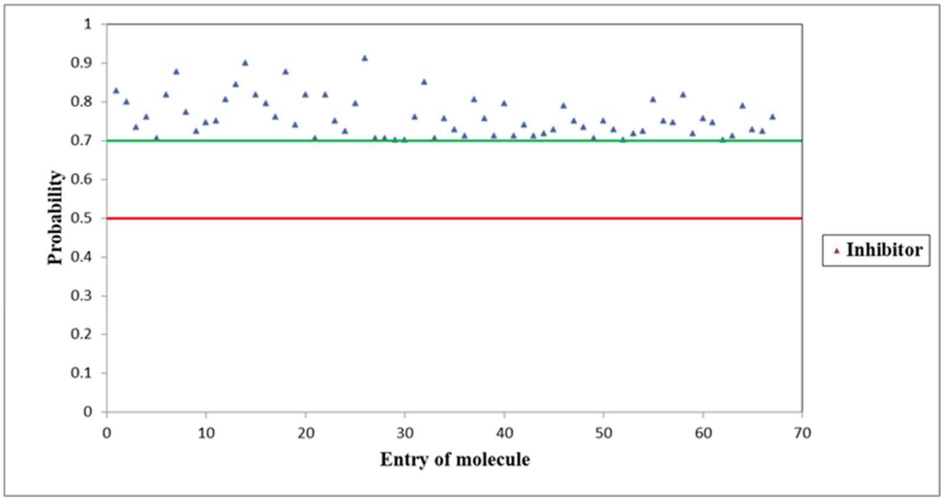

(B)

Figure S3. The visualized distributions of compounds. (A) in the external validation set (55 compounds), (B) in the date set of molecules singled out by virtual screening with RF scores greater than 0.7 (67 compounds).

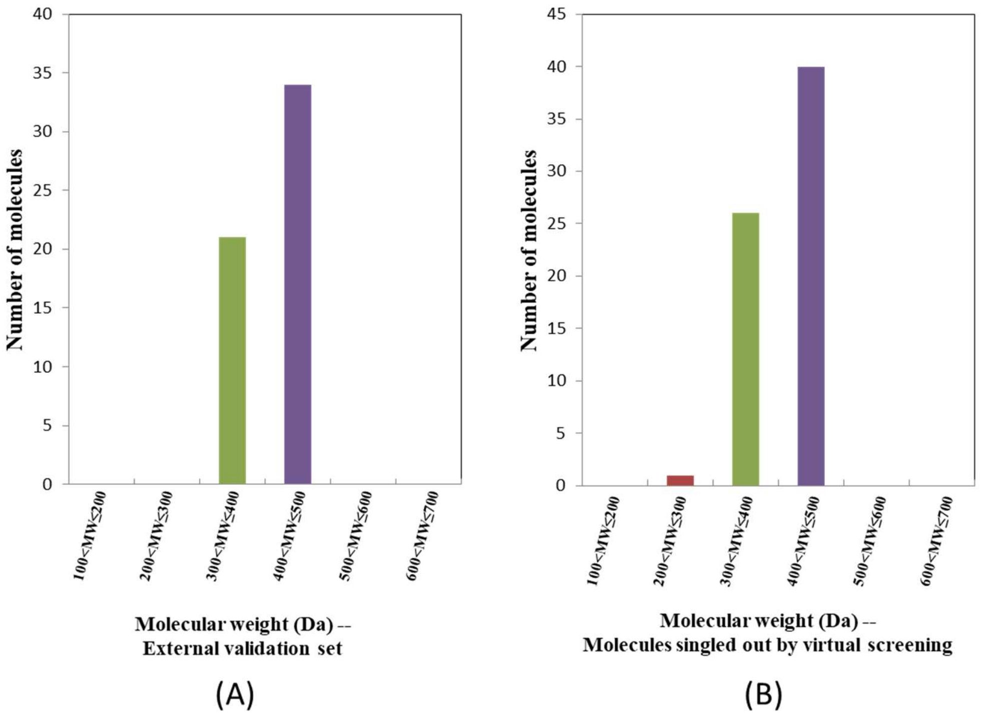

Figure S4. The molecular weight distributions of compounds. (A) in the external validation set (55 compounds), (B) in the date set of molecules singled out by virtual screening with RF scores greater than 0.7 (67 compounds).
